# Frontiers in New Drug Discovery: From Molecular Targets to Preclinical Trials

**DOI:** 10.3390/ijms24098321

**Published:** 2023-05-05

**Authors:** Dmitry Aminin

**Affiliations:** G.B. Elyakov Pacific Institute of Bioorganic Chemistry, Far Eastern Branch of the Russian Academy of Sciences, 690022 Vladivostok, Russia; daminin@piboc.dvo.ru

The intention of this Special Issue is to focus on new aspects of drug discovery, including the search for new molecular targets of various diseases, the creation of new modern methods for diagnosing diseases, the development of new test systems and kits for assessing the selectivity and effectiveness of new drugs, the study of the molecular mechanisms of biologically active compounds, the formulation of new drugs, pharmacokinetic and pharmacodynamic studies and preclinical trials of important molecules.

Most of the articles in this Special Issue are devoted to the creation of new compounds with an antitumor activity and for anticancer therapy and cancer diagnostics. Thus, new platinum (II) cationic five-coordinate complexes and their physicochemical properties were described. Their biological activity was investigated against two pairs of cancer and non-cancer cell lines. The tested drugs were internalized in cancer cells and were able to activate the apoptotic pathway [1]. The regio- and diastereoselective synthesis of novel pyrrolidine-fused spiro-dihydrophosphacoumarins were presented in [2]. This new approach in the synethesis of spiro-dihydrophosphacoumarins is complementary to existing approaches and provides an easy entry to the otherwise inaccessible derivatives, some of which have significantly greater cytotoxic activity against the HuTu 80 cell line than the reference 5-fluorouracil. 

Two articles consider the creation of phototoxic compounds and methods of photodynamic cancer therapy. Mikra et al. [3] synthesized a set of arylazo sulfones, known to undergo N–S bond cleavage upon exposure to light, and investigated their activity in the dark and upon the irradiation of DNA. It turned out that exposure to UV light leads to structural rearrangements in some arylazo sulfones, increasing their cytotoxic activity against several cell lines. Thus, the temporal and spatial manipulation of light may enable these new scaffolds to be useful for the creation of phototoxic pharmaceuticals. One study [4] is devoted to the development of a method of minimally invasive focal therapy to reduce the number of prostate tumor cells while maintaining delicate structures in the treatment of prostate cancer. Prostate tissue samples were treated with oxygenated solutions of Rose Bengal or protoporphyrin IX disodium salt and illuminated with visible light. They were then analyzed for changes in morphology using microscopy and assessed for measurements detected using MRI. The parameters recorded indicated a pronounced antitumor effect of the drugs after photodynamic exposure. 

In the investigation by Samir et al. [5], the expressions of a number of molecular markers characteristic of tumor-associated macrophages (TAMs) were localized. Immunohistochemical and morphometric analyses of tissue samples from patients with lung adenocarcinoma revealed that higher numbers of a basic leucine zipper transcription factor of macrophage (MAFB+) cells were significantly correlated with increased metastasis, a high recurrence rate, increased lymphatic permeability, higher vascular invasion, and pleural infiltration. It turned out that an increase in the number of MAFB+ cells is accompanied by the poor survival of tobacco-dependent patients, and MAFB can be considered a marker for TAMs and a prognostic biomarker for smokers with lung cancer. Using big data mining and deep learning methods, Yeh et al [6] studied the pathogenesis of two subtypes of diffuse large B-cell lymphoma. Applying a modern approach, the authors were able to compare the main signaling pathways and pathogenic mechanisms, making possible to identify pathogenic biomarkers as drug targets for two tumor subtypes. Through the use of a deep-neural-network-based drug-target interaction model that was trained in advance, assessments of the drug regulation ability and drug toxicity were carried out. As a result, the two drug combinations were proposed to alleviate two subtypes of B-cell lymphoma, respectively.

One publication examined the radioprotection activity of two lipid-lowering drugs, Pravastatin and Metformin, that are used to treat type 2 diabetes mellitus [7]. It was found that the combined administration of these two drugs had a therapeutic effect on acute radiation-induced intestinal injury in mouse and mini-pig models and markedly increased animal survival in a radiation-induced intestinal injury model. The effect was accompanied by a significant reduction in radiation-induced biochemical damages. 

Several articles concern the study of the anti-inflammatory activities and neuroprotective properties of chemical compounds and their antimicrobial potential and ability to inhibit key enzymes. Three novel regioisomeric analogues of naphthyl-*N*-acylhydrazone derivatives were studied with respect to their anti-inflammatory activity in in vitro and in vivo models of inflammation. The compounds did not have toxic properties. At the same time, they significantly reduced the migration of leukocytes associated with inflammation and the production of nitric oxide and interleukin-1β; however, they did not affect the activity of inducible nitric oxide synthase and did not demonstrate an NO scavenger effect. The data show that these substances have promising effects for the development of new drug prototypes [8]. The neuroprotective properties of a series of recombinant sea anemone peptides were studied in [9]. It was established that two selected peptides inhibit the formation of ROS and NO in Neuro-2a neuroblastoma cells induced by paraquat and rotenone to mimic Parkinson disease in vitro. Spectrofluorometry, fluorescence imaging and SPR analysis have shown that the peptide protective mechanism is mediated by the inhibition of the P2X7 receptor’s functionality. 

Using combined computer simulation methods, the antimicrobial properties of benzoic acid derivatives, sigmacidins, which inhibit the interaction of the bacterial RNA polymerase-σ factor and demonstrate pronounced antimicrobial activity against Streptococci, were studied. The use of the QSAR, docking and molecular dynamics methods made it possible to predict means of further optimizing the chemical structure of sigmacidins in order to enhance their antimicrobial properties and create new drugs against pathogenic *Streptococcus pneumoniae* [10].

Acetylcholinesterase inhibitors are widely used in medical practice. One review [11] collected information on natural inhibitors, the sources of their production, and the relationship of their chemical structures with therapeutic effects and described various methods for determining their biological activity. The review emphasized that further studies of the mechanisms of action and structure–activity correlations are needed to discuss the use of new cholinesterase inhibitors for the treatment of certain diseases.
